# An appraisal of traditional knowledge of plant poisoning of livestock and its validation through acute toxicity assay in rats

**DOI:** 10.3389/fphar.2024.1328133

**Published:** 2024-02-14

**Authors:** Faisal Rasool, Zaheer Ahmed Nizamani, Khawaja Shafique Ahmad, Fahmida Parveen, Shahzad Akbar Khan, Naveed Sabir

**Affiliations:** ^1^ Department of Veterinary Pathology, Sindh Agriculture University Tandojam, Hyderabad, Pakistan; ^2^ Department of Pathobiology, University of Poonch Rawalakot, Rawalakot, Pakistan; ^3^ Department of Botany, University of Poonch Rawalakot, Rawalakot, Pakistan

**Keywords:** plant poisoning, traditional knowledge, relative frequency of citation, fidelity level, acute toxicity, LD50

## Abstract

**Background:** Kashmir Himalaya hosts the most diverse and rich flora in the world, which serves as grazing land for millions of small ruminants in the area. While most plant species are beneficial, some can be poisonous, causing economic losses and animal health issues for livestock. Consequently, this study is the first comprehensive report on the traditional phyto-toxicological knowledge in District Muzaffarabad and the assessment of its authenticity through experimental studies in rats.

**Methods:** The data regarding traditional knowledge was gathered from 70 key respondents through semi-structured interviews, which was quantitatively analyzed and authenticated through plant extract testing on Wistar female rats and comparison with published resources.

**Results:** A total of 46 poisonous plant species belonging to 23 families and 38 genera were reported to be poisonous in the study area. Results revealed that leaves were the most toxic plant parts (24 species, 52.1%), followed by the whole plant (18 species, 39.1%), stem (17 species, 36.9%), and seeds (10 species, 21.7%). At the organ level, liver as most susceptible affected by 13 species (28.2%), followed by the gastrointestinal tract (15 species, 32.6%), nervous system (13 species, 8.2%), dermis (8 species, 17.3%), renal (7 species, 15.2%), respiratory (4 species, 8.7%), cardiovascular system (3 species, 6.5%), and reproductive system (2 species, 4.3%). The poisonous plant species with high Relative frequency citation (RFC) and fidelity level (FL) were *Nerium oleander* (RFC, 0.6; FL, 100)*, Lantana camara* (RFC, 0.6; FL, 100), and *Ricinus communis* (RFC, 0.6; FL, 100). Experimental assessment of acute toxicity assay in rats revealed that *Nerium oleander* was the most toxic plant with LD_50_ of (4,000 mg/kg), trailed by *Ricinus communis* (4,200 mg/kg), *L. camara* (4,500 mg/kg), and *Datura stramonium* (4,700 mg/kg); however, other plants showed moderate to mild toxicity. The major clinical observations were anorexia, piloerection, dyspnea, salivation, tachypnea, constipation, diarrhea, tremor, itchiness, and dullness.

**Conclusion:** This study showed that numerous poisonous plants pose a significant risk to the livestock industry within Himalayan territory, leading to substantial economic losses. Consequently, it is of utmost importance to conduct further comprehensive studies on the phytotoxicity of plants.

## Introduction

Plants serve as the primary food source for herbivores and are also used for the treatment of many diseases (Elshafie et al., 2023). Nonetheless, some plants are also known for their toxicity in ruminants due to the presence of toxic compounds (Dubey et al., 2018). These toxins can have detrimental effects on the ruminants, leading to economic losses such as health deterioration, low productivity, deformed offspring, and even death (Kebede et al., 2015; Paim et al., 2023).

Ethnoveterinary studies involve the exploration and documentation of traditional knowledge and practices related to using plants, minerals, and other natural resources for managing animal health and wellbeing (Teuscher and Lindequist, 2023). These studies have gained significance due to their potential to offer sustainable and cost-effective solutions for livestock healthcare in rural and resource-limited areas. However, alongside the benefits, it is essential to address the potential risks associated with the use of plant-based remedies, including the risk of poisoning (Uprety et al., 2022).

Although the livestock are selective feeders while grazing or browzing in rangelands, occasional livestock poisonings are widespread worldwide in free-range systems due to poisonous plants (McKenzie, 2020). These cases may occur when preferred plant species are scarce during dry seasons, forcing animals to browse perennial shrubs and bushes rich in toxic secondary metabolites (Oladosu et al., 2016), causing production loss, morbidity, and mortality in animals (Dubey et al., 2018). The poisonous plants may sometimes be eaten accidentally (Nielsen et al., 2019). In addition, nomads and villagers may not be familiar with potentially toxic plants during hay making (Majeed et al., 2020). Thus, the most important factors that expose ruminants to plant poisoning are feed shortage, nutritional deficiency, and sudden exposure to plants (Stegelmeier et al., 2013).

Plant toxins might be synthesized within the plant itself, produced by microorganisms associated with animals, or simply incorporated and accumulated by the plant (Onyeyili et al., 2018). Plants can be categorized into different groups based on their poisonous principle (Oladosu et al., 2016). These toxic properties tend to be chemically identical or even similar within a single group of related plant species (Botha and Penrith, 2008), the nature of these toxins changes with respect to their origin and surrounding environmental conditions (Desta, 2019). The primary toxic compounds that serve as defense mechanisms in plants against herbivores include tannins (Kara and Sürmen, 2019), phenolics (Stevenson et al., 2017), alkaloids (Matsuura and Fett-Neto, 2015), phytohemagglutinins (Dubey et al., 2018), terpenes (Dearing and Weinstein, 2022), cyanogenic glycosides (Gershenzon, 2017), and oxalates (Karabourniotis et al., 2020).

Plant poisoning has the potential to impact a wide range of body systems. Numerous factors contribute to the toxicity of plants in livestock (Botha and Penrith, 2008). Among ruminants, the severity of plant toxicity can differ based on the animal species or breed, age, gender, and overall health status (Leong et al., 2017). Further, plant growth conditions, developmental stages, specific parts of the plant, and consumption amounts are all critical factors that influence the outcome (Dixit, 2007). The poisoning is also influenced by various chemical factors like size of particles, solubility, toxicity, rate of absorption and excretion, tendency to bind with body tissues or fluids, and absence of established metabolic processes (Khan et al., 2018). Diagnosis of plant-induced livestock poisoning involves considering the animals’ history, observed clinical symptoms, *postmortem* findings, signs of plant consumption through grazing or browsing, and the presence of toxic plants in the gastrointestinal tract (Botha and Penrith, 2008).

The harmful effects of poisoning can appear in ways, such as through contact, ingestion, absorption, and inhalation (Khan et al., 2018). Animals that encounter plants may experience symptoms. For example, they might suffer from skin irritation if they touch the plants (Modi et al., 2009), or they could be internally poisoned if they consume them (Crosera et al., 2009). There is a large diversity of plant toxins that can harm body systems in myriad ways. Aristolochic acid found in *Aristolochia fangchi* S.M.Hwang (Nortier et al., 2000) and *Aristolochia clematitis* L. has carcinogenic properties (Gluhovschi et al., 2010). Those who consume herbal remedies laced with *A. fangchi* or animals that consume crops laced with *A. clematitis* suffer from fatal urothelial neoplasms (Nortier et al., 2000). Ricinol, which is derived from *Ricinus communis* L. is responsible for a variety of symptoms, including gastrointestinal irritation, anorexia, apathy, dyspnea, piloerection, abortion, and acute purgation (Khan et al., 2018). Lantadenes from *Lantana. camara* L. cause hepatotoxicity, chronic cholestasis, piloerection, and photosensitivity (Khan et al., 2018). *Leptopus cordifolius* Decne. has a major toxin known as cicutoxin that causes muscle weakness, piloerection, bone lesions, aneurysms, and burning sensations (Rahman et al., 2021). The *Datura stramonium* L. contains sopolamine and hyoscyamine, which cause dermatitis, polydipsia, mydriasis, anorexia, abdominal pain, stupefaction, and restlessness (Murtala et al., 2023). Oleandrin from *Nerium oleander* L. causes a variety of effects, including piloerection, stomach salivation, vomiting, diarrhea, irregular heartbeats, drowsiness, tremors, seizures, seizures, coma, hepatotoxicity, and nephrotoxicity (Abdou et al., 2019). The cardiotoxic effect of *Nerium oleander*, for example, may cause death by severe cardiac arrhythmias (Shridhar, 2022).

The Muzaffarabad division in Azad Kashmir is rich in plant diversity, including a wide variety of potentially toxic plant species. These plants can be a threat to both animal health and the livestock industry in the region. Prior to our research, no comprehensive study had been conducted to identify and describe the native poisonous plants in Azad Jammu and Kashmir. Therefore, this study represents a pioneering effort to bridge this research gap by identifying these poisonous plants and thoroughly examining the harmful impacts of plant poisoning on animal wellbeing. The information regarding indigenous poisonous plants was collected from livestock holders through periodic surveys and validated through existing literature. Additionally, the accuracy of knowledge held by livestock keepers and available in published reports was assessed by testing the toxicity of selected poisonous plants through acute toxicity assay in Wistar female rats.

## Materials and methods

### Ethics approval and consent to participate

Formal consent was received from informants regarding data collection and publication, and the Participatory Rural Appraisal (PRA) approach, as mentioned in the Kyoto Protocol, was applied with the consent of the informant. Additionally, the study was approved by the Ethical committee and Board of Advance Studies and Research, Sindh Agriculture University Tandojam, in its 144th meeting under serial number DAS/937/2022.

### Study area

**FIGURE 1:**
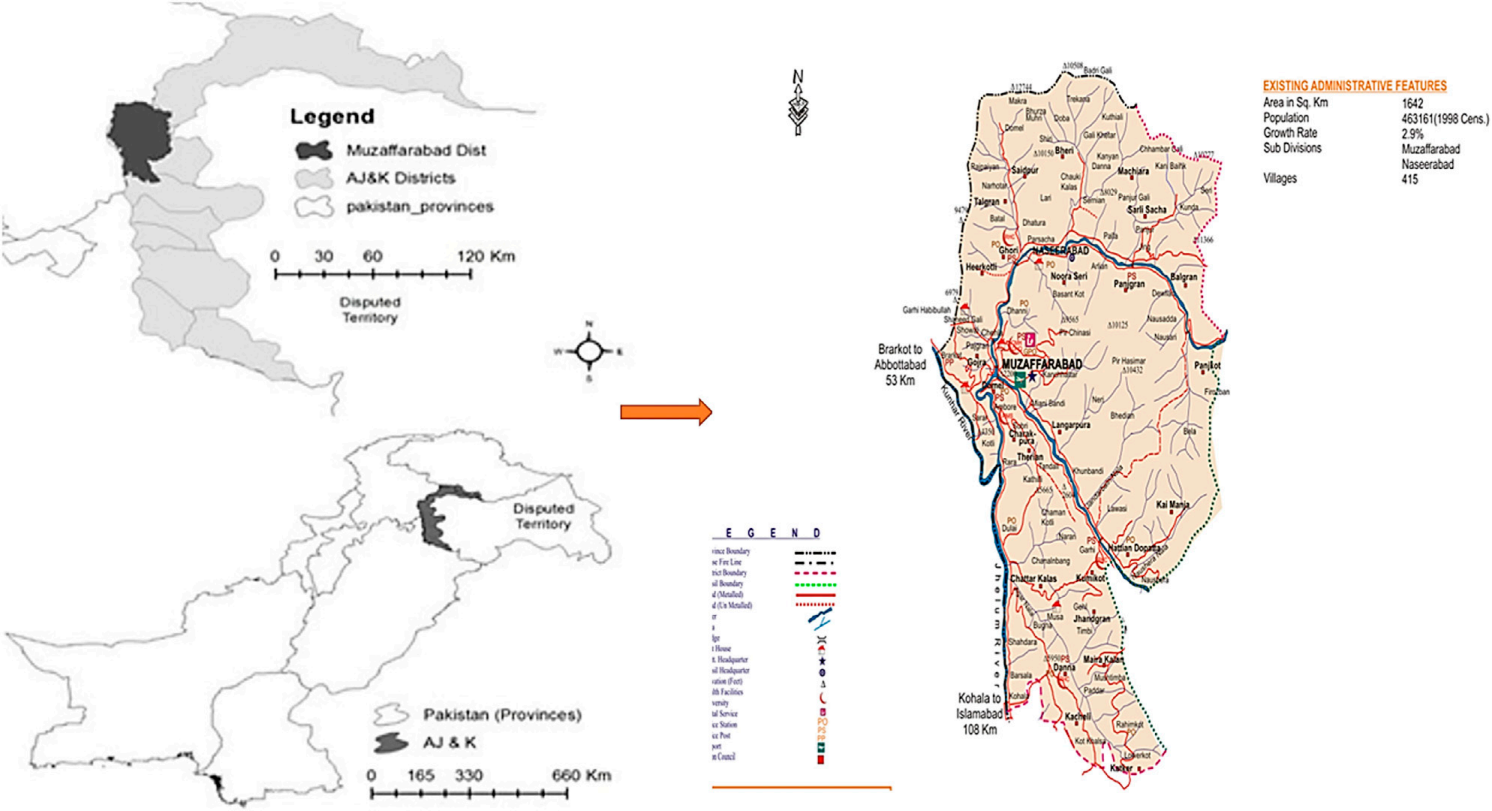
Map of the study area showing study sites.

Muzaffarabad is the capital of Azad Jammu and Kashmir, Pakistan (Figure 1). It is located in the western Himalayan range, between latitude 34º03′-34º35′N and longitude 73º23′-73º45′E, with a land area of around 1,642 km^2^. The study area exhibits a wide range of elevations, with the lowest point located in the southern Kohala locality at 582 m above sea level (m. a.s.l.) The highest points in the region are found at the summits of Makra Mountain and Neela Ganja Mountain, which reach heights of 3,819 m and 4,473 m, respectively. The variation in elevation across the study area has important implications for a range of ecological processes, including temperature gradients, precipitation patterns, and vegetation distribution. It also significantly impacts human activities such as agriculture and transportation infrastructure.

The bordered by the Muzaffarabad district with Hattian Bala district to the east, Neelum district to the northeast, Hazara division in Khyber Pakhtunkhwa (KPK) to the north and northwest, Bagh district to the south, and Murree hills in Punjab to the southwest. The topography is distinguished by a scenic landscape, with mountains ranging from subtropical valleys to the fascinating alpine zones of the Himalayas. Natural beauty abounds in the region, with dense forests, swiftly flowing rivers, and meandering streams. The Jhelum and the Neelum are the two notable rivers in the area (Khan et al., 2018).

The climate in Muzaffarabad district is subtropical highland. Temperatures range from −2–40°C between January and August. The average annual rainfall ranges between 1,000 and 1,300 mm, with the majority falling between May and August (around 680 mm). Winds blow from west to east during the day and from southeast to north at night. The wind speed is higher in the afternoon than in the morning. The snow line is approximately 1,200 m above sea level in winter and approximately 3,300 m. a.s.l. in summer (Qasim et al., 2010; AJK, 2015).

### Survey for ethnoveterinary data collection and preservation

A thorough survey was conducted between August 2018 and January 2021 to gather data on livestock species, including goats, cows, sheep, buffaloes, and camels. The primary goal of the survey was to catalog and gather information on poisonous species and the risks involved. In 20 different places throughout the Muzaffarabad division, a total of 70 interviews with local herders and farmers were conducted. An observational field walk was included in the interviews, which used a semi-structured questionnaire. 40 skilled women and 30 highly experienced men, all between the ages of 30 and 65 years, participated in the study and had in-depth knowledge of raising livestock. The interviewers were an important part of the team that helped collect and identify plant specimens. The informants were chosen using the snowball method, and they came from various backgrounds and occupations, including farmers, livestock owners, veterinary professionals, and nomads (Bakarwals). Each subject gave informed consent prior to the interview, and the study complied with the International Society of Ethnobiology’s Code of Ethics (Mattalia et al., 2020).

Local names, usage, availability, and other valuable information about the plants, including toxic plant parts and plant toxicity levels, the type of animal affected, the nature of the disease, the type of symptoms that manifested, etc., were recorded on-site during the interviews. The plant species were collected, identified, noted, dried, pressed, and mounted on typical herbarium sheets. Plant identification was done by Dr. Sajjad Hussain, Assistant Professor and curator of the Poonch Herbarium, Department fo Botany, University of Poonch Rawalakot. Additionally, the *Flora of India* (https://sites.google.com/site/efloraofindia/), the *Flora of Pakistan* (Ali and Nasir, 1980), as well as the World Flora Online (https://www.worldfloraonline.org/) were used in the identification process. For the voucher specimens, standard herbarium processes were strictly followed. The voucher samples were kept in the University of Poonch Herbarium in the Department of Botany, University of Poonch Rawalakot, for future use.

### Quantitative analyses of survey data

#### Relative frequency citation (RFC)

The data gathered was subjected to quantitative analysis using the Index of RFC, employing the formula provided below:

$$\text{RFC}=\text{FC}/N\;\left(0\;<\text{ RFC }<\;1\right)$$

The RFC functions as a gauge for the particular importance of each species, gauged by how often they are mentioned in citations. The FC represents the proportion of survey participants who brought up the use of a specific species, divided by the total number of participants (N). It is worth mentioning that the analysis does not consider the various ways the species are used (Majeed et al., 2020).

#### Fidelity level (FL)

FL was assessed for frequently mentioned diseases or conditions using the approach outlined by Hoffman and Gallaher (2007). This indicates the percentage of individuals who identified a particular species as the cause of poisoning compared to the overall number of individuals who acknowledged the role of plant poisoning in affecting livestock (Khunoana et al., 2019).

$$\text{FL}=\left(\text{Np}/N\right)\;*\;100$$

In this context, Np is the count of individuals who associated a particular livestock ailment with plant poisoning, whereas N represents the overall count of individuals who witnessed disease symptoms or indicators in their livestock due to plant poisoning.

#### Priority ranking test

A priority ranking test was used to rank the top poisonous plants. In this test, 15 selected key respondents were asked to rank the plants based on the toxicity in the study area by following a method of Martin (1995).

#### Validation of folk knowledge through published scientific literature

**FIGURE 2:**
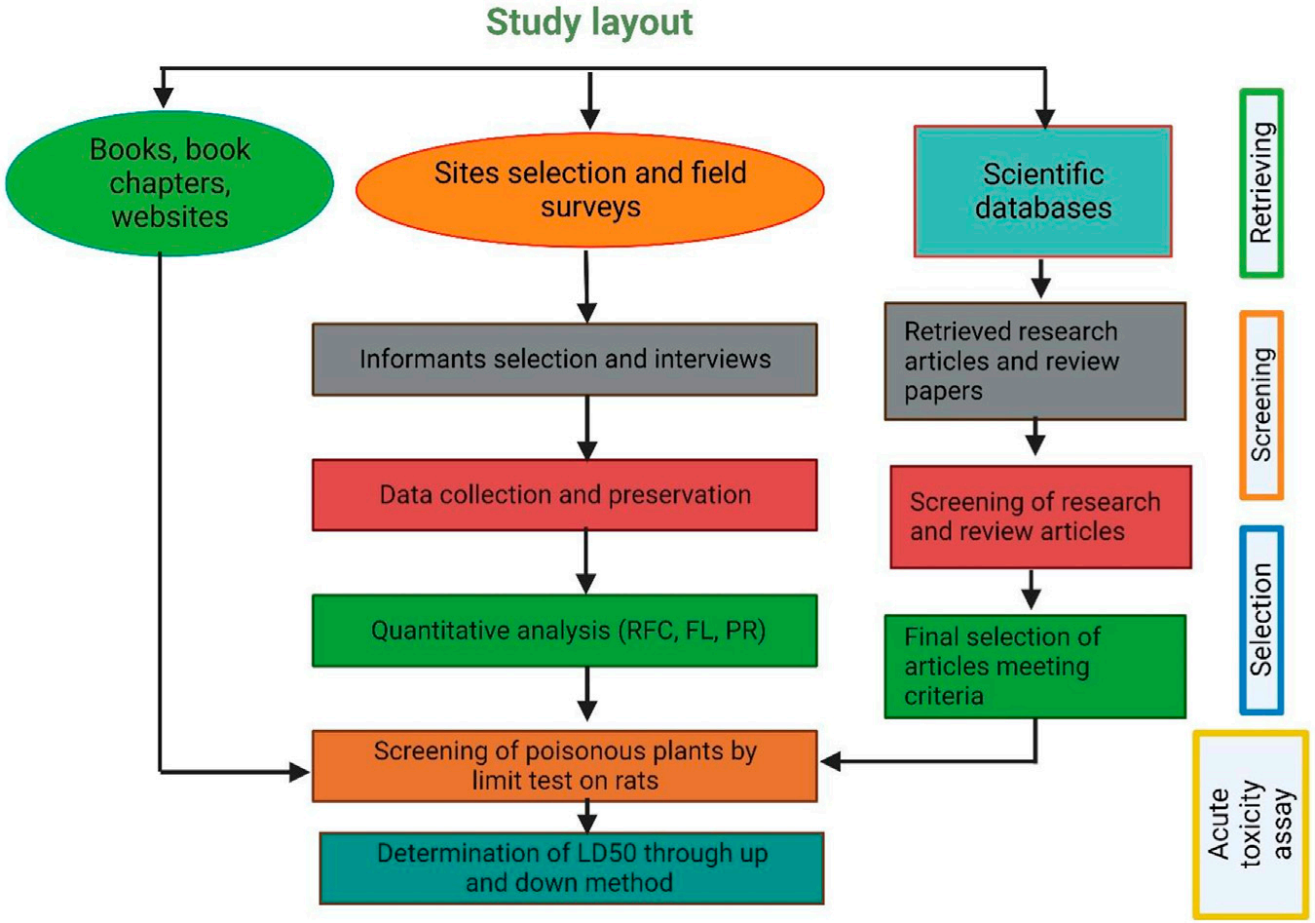
Flow chart showing how information was retrieved from different resources.

For authenticity of the data obtained from informants on poisonous plants, a comprehensive literature review was conducted by systematically analyzing research papers from various electronic-based journals and books reporting information related to poisonous plants, their toxicity, and the symptoms of toxicity (Figure 2). Multiple search engines were utilized for a literature search, including Web of Science, Google Scholar, ResearchGate, PubMed, SciFinder, Scopus, Chemical Abstracts Services, books, dissertations, and technical reports. The search was performed using specific keywords, such as “poisonous plants,” “lethal dose LD_50_,” and “toxic compounds."

In every research article, we gathered information about various characteristics of each plant. These included their scientific names, family classification, names used locally, lifeform, the specific toxic plant parts, type of disease and animal affected, recommended dosage and levels of toxicity, specific toxin, and mechanism of action, clinical symptoms, susceptible organ and species.

Only those articles were selected that presented original research, were written in English, had undergone a peer-review process, and had full text available online through various scientific search engines. A total of 129 documents were ultimately selected for inclusion in this review, covering the period from 1981 to March 2023. In addition to academic research articles, this review’s basic framework was put together by consulting a range of books about local plants, broadcast news sources, and print media. Online botanical resources such as www.theplantlist.org, www.plantoftheworldonline.org, www.gbif.org, www.jstor.org, and www.tropics.org (accessed on 18 June 2023) were also used to gather data on other names for plants and their geographic distribution.

## Assessment of acute toxicity of selected poisonous plants

### Poisonous plant selection and preparation of extracts for acute toxicity assays

The 12 poisonous plants for toxicity assays were selected on the basis of a ranking based on a literature review, a survey from stakeholders, and the abundance of plants in the selected area. Poisonous plant leaves were collected from various areas in Muzaffarabad. Leaves were shade-dried at room temperature, and an electrical homogenizer was used to grind them to a powder. The ratio of the dried part’s (1.5 kg) weight to the original weight (4 kg) was calculated and then kept in glass bottles for further use in the experiment. The 120 g powder material was poured into a transparent glass bottle with 800 mL of 95% ethanol and soaked for 3 days. The glass bottle was labeled with a plant name. Further, the soaked material was filtered using a muslin cloth or filter paper. A rotary evaporator was used to remove the solvent (ethanol) from the flask. However, the extract was dried more thoroughly in the hot air oven at 65°C and stored at 40°C.

### Selection and housing of animals (rats) for the experiment

Wistar female rats, aged 8–12 weeks, weighing 125–150 g, were obtained from DOW University of Health Sciences (DUHS), Karachi. These rats were maintained in the animal house of the Department of Veterinary Parasitology, Sindh Agriculture University, Tandojam. The animals were maintained under standard environmental conditions of a temperature of 24°C ± 10°C, a 12 h dark-light cycle, free access to drinking water, and a standard pelleted diet.

### Screening of selected plants for toxicity by limit test

The limit test was performed as per guidelines of the Organisation for Economic Cooperation and Development (OECD) 423. The purpose of the limit test was to determine the toxicity of themostly reported plants, selected on the basis of the survey and literature review, by initially testing a dose of 2,000 mg/kg body weight and, as no deaths occurred in rats, finally a higher dose of 5,000 mg/kg body weight was tested showing higher signs of toxicity.

### Preparation of doses and administration of treatments

The animals were weighed before the experiment (Table 1), and doses were calculated as per body weight. The ethanol extract of each plant was weighed, dissolved in 0.1 mL dimethylsulfoxide (DMSO), and then water was added to make a final volume of 1 mL. Rats were deprived of food and water 2 h before the experiment. The doses were orally administered to rats with the help of a 16-gauge gavage needle. The animals were examined for mortality and apparent changes at specific intervals, including the first, sixth, 12th, and 24th hour. Subsequently, the animals were monitored once a day for 2 days.

**TABLE 1:** Experimental design for determination of plant toxicity by Limit test showing different groups of animals orally administrated with different concentrations of extract (treatments).

Groups	Treatments
Group 1: Control	Water
Group 2: Sham control (DMSO + water)	10% in water
Group 3: Plant extract	2,000 mg/kg body weight
Group 4: Plant extract	5,000 mg/kg body weight

Initially, for testing the dose of 2,000 mg/kg body weight, 36 rats were divided into 12 groups, each having three rats. Regular tap water was given to the control group, while 10% DMSO was added to water for the sham control group. The remaining treatment groups received a single oral dosage of various plant extracts (2,000 mg/kg body weight), as shown in Table 1. Following the OECD’s recommendations, the experiment was repeated using a new set of three rats for every different plant extract because no fatalities were observed, as outlined in Table 2.

**TABLE 2:** Classification of toxicity of substances based on LD_50_ and dose ranges according to Organization for Economic Cooperation and Development (OECD) guidelines (Teke and Kuete, 2014).

LD_50_ (mg/kg)	Category
1 or less	Extremely toxic
1–50	Highly toxic
50_500	Moderately toxic
500_5,000	Slightly toxic
5,000_15,000	Practically non-toxic
More than 15,000	Relatively harmless

Next, the 5,000 mg/kg body weight dose was tested. For this experiment, 30 rats were divided into 10 groups, each having three rats. The control group received regular tap water, while the sham control group was given 10% DMSO in water. Plant extracts (5,000 mg/kg body weight) were administered as a single oral dose for the remaining treatment groups. If the first animal survived, two further animals were dosed. If only one of the three animals died, the LD_50_ value was expected to exceed 5,000 mg/kg. If both animals died, then dosing proceeded at 2,000 mg/kg. If all three animals survived, the maximum dose administered was considered to be the LD_50_ value.

### Determination of lethal dose 50 (LD_50_) through up-and-down method

Plant extracts causing mortality in rats at the dose of 5,000 mg/kg body weight were further tested using the up-and-down procedure following OECD test guidelines 425 (OECD, 2001) to determine the LD_50_. For this purpose, doses of 2,500, 3,000, 3,500, 4,000, 4,500, and 5,000 mg/kg were applied, respectively. The plants were classified into various toxicity grades as per the toxicity standards of OECD (Table 2). LD_50_ below 5,000 mg/kg is classified as highly toxic, while LD_50_ above 5,000 mg/kg is considered harmless (Ea et al., 2021).

The LD_50_ was calculated using the arithmetic method by (Alui and Nwude, 1982) using the formula LD_50_ = LD_100_-(Dd × Md)/N; where LD_100_ = dose that caused 100% mortality; N = number of animals per group; Dd × Md = dose difference (Dd) multiplied by mean death (Ea et al., 2021). LD_50_ is considered highly toxic if it is below 2,000 mg/kg, whereas it is considered safe if it exceeds 5,000 mg/kg (Hayes and Loomis, 1996).

### Data analysis

The results were presented as the averages and the standard deviation (SD). The statistical significance was assessed through a one-way analysis of variance (ANOVA) followed by the *post hoc* least-significant difference (LSD) test. Results with *p*-values lower than 0.05 were regarded as significant.

## Results

### Demography

To collect data on plant poisoning affecting livestock across different areas of Muzaffarabad district, 70 participants were interviewed, consisting of 30 males and 40 females. This group encompassed various roles, including Gujjars (farmers), Bakkarwals (nomads), laboratory professionals, veterinarians, livestock holders, artificial inseminators, dairy owners, milk vendors, traditional healers, and agricultural experts. Notably, all traditional healers and veterinarians were of the male gender. The age spectrum of the respondents ranged from 20 to 80 years. Among the respondents, the predominant proportion, approximately 67.14%, lacked formal education, while the remaining individuals held masters or Ph.D. degrees (Table 3).

**TABLE 3:** Demographic profile of the selected informants from the study area showing their gender, categories, and educational background percentages.

Variable	Categories	No. of persons	Percentage
Gender	Male	30	42.86
	Female	40	57.14
	30–44 years	20	28.57
	45–60 years	25	35.71
	Greater than 60 years	25	35.71
Informant categories	Practitioners	10	14.29
	Nomads	20	28.57
	Veterinary doctors	07	10.00
	Farmers	13	18.57
	Cattle holders	15	21.43
	Livestock assistants	05	7.14
Educational background	Illiterate	48	68.57
	Literate	22	31.43

### Floristic contribution

**FIGURE 3:**
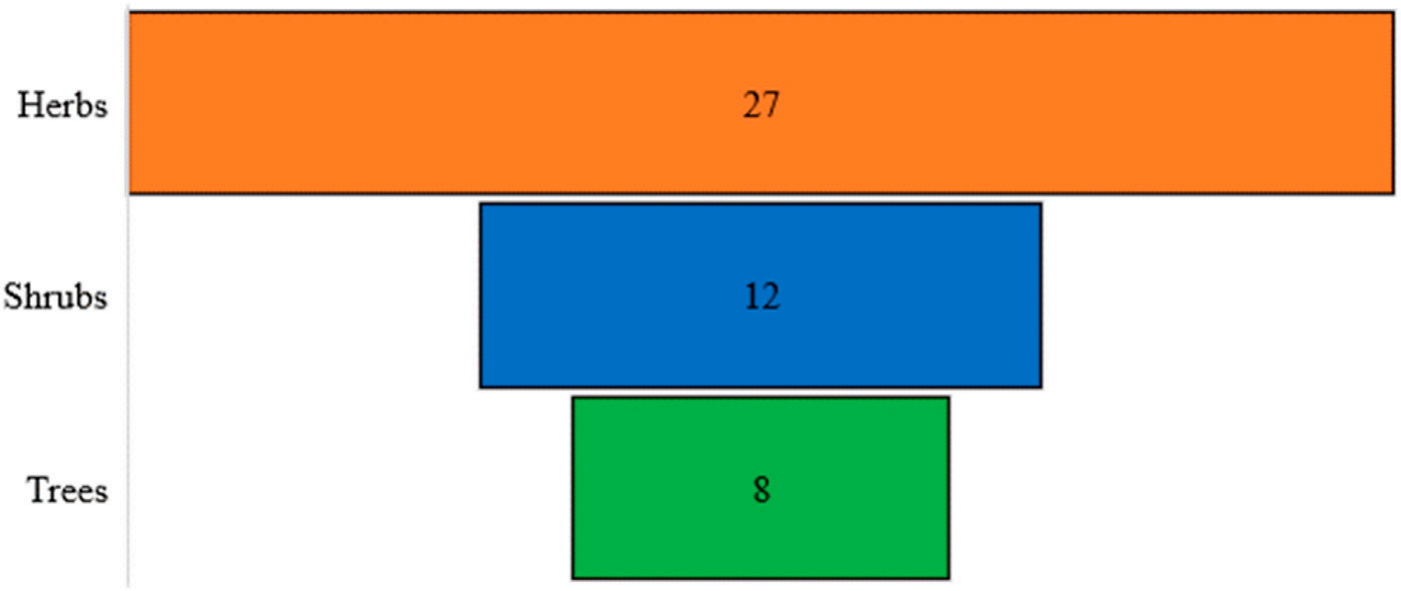
Floristic diversity showing lifeform spectra of the study area.

**FIGURE 4:**
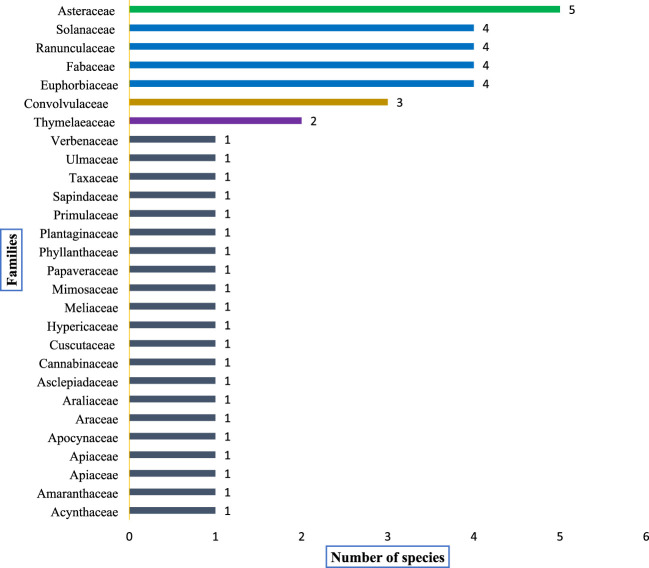
Shows dominant plant families and the distribution (number) of poisonous plants within each family recorded from the study area.

A total of 46 poisonous plant species belonging to 23 distinct families and 38 genera were documented (Table 4). The most prevalent life forms were herbs (26 species, 57.8%), followed by shrubs (12 species, 25.5%) and trees (8 species, 17.0%), as illustrated in Figure 3. Among these species, the most common family was Asteraceae (5 species, 10.8%), followed by Ranunculaceae (4 species, 8.7%), Solanaceae (4 species, 8.7%), Fabaceae (4 species, 8.7%), Euphorbiaceae (4 species, 8.7%), and Convolvulaceae (3 species, 6.5%). The remaining families were represented by either one or two species each (Figure 4).

**TABLE 4:** Enlisted poisonous plants with their toxic parts, susceptible animals, affected organs, and their phytotoxicity reports by informants.

Family	Botanical name	Voucher no.	Vernacular name	Life form	Toxic part	Susceptible animals	Affected organ	Phytotoxicological claims
Acanthaceae	*Justicia adhatoda* L	POONCH-4	Bhekar	Shrub	Leaves, seeds	Rodents, rabbits, canine	Lungs	Anxiety, lowering of blood sugar, inflammation of the intestine
Amaranthaceae	*Achyranthes aspera* L	POONCH-1	Puthkanda	Herb	Whole plant	Rodents	Central nervous system	Loss of weight, appetite, dehydration, piloerection, foot disease, sluggishness
Apiaceae	*Conium maculatum* L	POONCH-2	Shokra	Herb	Whole plant	Small ruminants, horses, avians	Central nervous system	Shivering, loss of appetite, rapid heartbeat and respiration, muscular pain, salivation, urination, convulsions, coma, and death
Apiaceae	*Heracleum canescens* Lindl	POONCH-3	Hermal	Herb	Whole plant	Domestic animals	Liver	Dermatitis, hypersensitivity
Apocynaceae	*Nerium oleander* L	POONCH-5	Kanair	Shrub	whole plant, stem	Wild and domestic animals, rabbit, and monkey	Cardiac, hepatic, renal, lungs, intestine, and skin	Bulging of eyeballs, stomach pain, salivation, emesis, motion, piloerection, irregular heartbeat, weakness, sluggishness
Araceae	*Alocasia indica* (Lour.) Spach	POONCH-6	Lambapatar	Herb	Leaves	Rodents	Hepatic, testis	Loss of hormones (LH and FSH), infertility, weight loss, dysfunction of kidney and liver
Araliaceae	*Hedera nepalensis* K.Koch	POONCH-7	Karera	Shrub	Whole plant	Cattle, buffalo, small ruminants, and rodents	Skin, stomach	Inflammation of skin, acute purgation, loss of movement, death
Asclepiadaceae	*Calotropis procera* (Aiton) W.T.Ation	POONCH-8	Desi aak	Shrub	Latex, root, stem, leaves	Rodents, rabbit	Hepatic, intestine, and cardiac	Tremors, irritation of skin, loss of pregnancy, difficulty in breathing, purgation
Asteraceae	*Ageratum conyzoides* L	POONCH-11	Neeli jari	Herb	Aerial parts	Rodents, guinea pigs, rabbits and canine	Cardiac, hepatic, renal	Severe itching, shivering, loss of appetite, hyperthermia, production of bitter milk, intensive motions, and death
Asteraceae	*Bidens bipinnata* L	POONCH-9	Kandili jari	Herb	Fruits	Rodents, avians	Gastrointestinal tract	Inflammation of liver and throat
Asteraceae	*Parthenium hysterophorus* L	POONCH-13	Gajar Botee	Herb	Whole plant	Domestic animals	Central nervous system, skin	Skin allergy, inflammation and rashes on skin, shivering, motion, breathing disorder, loss of appetite
Asteraceae	*Senecio vulgaris* L	POONCH-10	Peeli jari	Herb	Whole plant	Domestic animals	Hepatic	Reduction in milk production, jaundice, general weakness and death
Asteraceae	*Xanthium indicum* J.Koenig ex Roxb	POONCH-12	Jojra	Herb	Whole plant	Cattle, rabbits, canines, small ruminants, and horses	Hepatic, renal	Skin diseases, cramping, lowering of glucose level in blood, shivering, nausea, fever, coma, and death
Cannabinaceae	*Cannabis sativa* L	POONCH-14	Bhang	Herb	Whole plant	Companion animals	Central nervous system	Vomiting, anorexia, drowsiness
Convolvulaceae	*Convolvulus arvensis* L	POONCH-15	Hiran khuri	Herb	Whole plant	Domestic animals, rodents, rabbits, and canine	Gastrointestinal tract	Motion, gastrointestinal irritation, urinary disorders
Convolvulaceae	*Ipomoea carnea* Jacq	POONCH-16	Bilaiti aak	Shrub	Leaves	Rodents, guinea pigs, rabbits, and canine	Gastrointestinal tract	Blisters in the mouth, excessive salivation, acute diarrhea, shivering
Convolvulaceae	*Ipomoea purpurea* (L.) Roth	POONCH-17	Kharpoay	Herb	Seeds	Rodents, rabbits, dogs	Gastrointestinal tract	Gastrointestinal disorders
Cuscutaceae	*Cuscuta reflexa* Roxb	POONCH-18	Kaandal	Herb	Whole plant	Cattle, small ruminants, horses, poultry	Gastrointestinal tract	Nervousness, emesis, loss of appetite, cramping, purgation, abortion
Euphorbiaceae	*Mallotus philippensis* (Lam.) Müll.Arg	POONCH-22	Kumkum	Shrub	Seeds, root, stem, leaves, latex	Rodents, rabbits, canine	Skin	Milky juice of plant eyes and skin inflammation
Euphorbiaceae	*Euphorbia helioscopia* L	POONCH-20	Dudhal	Herb	Whole plant, latex	Rodents, chicken embryo	No lesions on vital organs, and normal architecture	Acute dermatitis when animals encounter milky sap, piloerection, frothing in mouth and throat, purgation, and weight loss
Euphorbiaceae	*Euphorbia heterophylla* L	POONCH-21	Dudli booty	Herb	Whole plant	Cattle, buffalo, small ruminants, Rodents	Hepatic, renal, central nervous system, and muscles	General weakness, low production of milk
Euphorbiaceae	*Ricinus communis* L	POONCH-19	Areni	Tree	Seeds, root, stem, leaves	Domestic animals, Rodents, guinea pigs, rabbits, canine	Gastrointestinal tract	Weakness, anorexia, apathy, dyspnea and moderate fever, abdominal cramps, piloerection, abortion, neurological signs, depression, acute purgation, gastrointestinal irritation
Fabaceae	*Bauhinia variegata* L	POONCH-23	Kachnaar	Tree	Seeds, leaves	Rodents	Gastrointestinal tract	Change in animal behavior, loss of immunity
Fabaceae	*Dalbergia sissoo* Roxb. ex DC.	POONCH-24	Shesham tali	Tree	Root, stem, leaves	Small ruminants, Rodents	Reproductive system	Loss of pregnancy, infertility
Fabaceae	*Lathyrus odoratus* L	POONCH-25	Matar	Herb	Whole plant	Small ruminants, Rodents, rabbit	Gastrointestinal tract	Emesis, weak bones, body pain
Fabaceae	*Melilotus indicus* (L.) All	POONCH-26	Peele singi	Herb	Whole plant	Horse, Rodents	Hepatic, hematology	Loss of blood, liver disorder, shivering, nervousness
Hypericaceae	*Hypericum perforatum* L	POONCH-27	Peela phool	Herb	Leaves	Domestic animals	Central nervous system	Inflammation of skin, photosensitization, cough, trembling of limbs, tramping
Meliaceae	*Melia azedarach* L	POONCH-28	Dareek	Tree	Fruit, leaves	Rodents, sheep, pigs	Central nervous system	Nervous and abdominal disorders
Mimosaceae	*Leucaena leucocephala* (Lam.) de Wit	POONCH-29	Lasni	Tree	Leaves	Rodents, guinea pigs, rabbits, dogs	Skin, gastrointestinal tract	Increased blood pressure and heart rate, sweating and vomiting
Papaveraceae	*Argemone mexicana* L	POONCH-30	Pili kandiar	Herb	Seeds	Rodents	CNS	Diarrhea, oedema of legs and feet
Phyllanthaceae	*Leptopus cordifolius* Decne	POONCH-31	Kurkani	Shrub	Leaves	Rodents, rabbits, canine	Hepatic, renal, pancreas	Lesion on bones, piloerection, muscular pain, aneurysms, paresthesia
Plantaginaceae	*Digitalis purpurea* L	POONCH-33	Lomar	Herb	Whole plant	Cattles, small ruminants		Gastrointestinal tract disturbance, weight loss, cramping, emesis
Primulaceae	*Anagallis arvensis* L	POONCH-32	Kokoon	Herb	Seeds	Rodents, calves, sheep	Renal, gastrointestinal tract	Dysentery, diarrhea, shivering of limbs, nausea, dyspnea, anorexia, nervousness, death
Ranunculaceae	*Aconitum heterophyllum* Wall. ex Royle	POONCH-35	Patrees	Herb	Flowers, leaves	Cattle, small ruminants	Dermatitis	This plant may cause skin inflammation and injury of mucous membranes
Ranunculaceae	*Caltha palustris* L	POONCH-36	Daldali genda	Herb	Leaves	Livestock, rats, rabbits, horses	Gastrointestinal disorder, diarrhea	Burning of the throat, vomiting, bloody diarrhea and gastric illness
Ranunculaceae	*Clematis grata* Wall	POONCH-34	Baili	Shrub	Leaves	Cattle, canines	Lungs	Gastrointestinal disorder, diarrhea
Ranunculaceae	*Ranunculus arvensis* L	POONCH-37	Butter cup	Herb	Leaves, seeds	Livestock, rats, rabbits, horses	Dermatitis	Fresh leaf juice causes cracks, itching and sores in the skin of humans and animals
Sapindaceae	*Dodonaea viscosa* Jacq	POONCH-42	Sanatha	Shrub	Leaves	Small ruminants, Rodents	Stomach	Nausea, diarrhea, shivering
Solanaceae	*Datura innoxia* Mill	POONCH-38	Indian apple	Herb	Leaves, seeds	Small ruminants, Rodents	Skin	Contact with the leaves causes several skin problems. Unintentional consumption of the seeds by animals causes dryness and sensation of the mouth and throat, stomachache, numbness, anorexia, mydriasis, polydipsia and restlessness
Solanaceae	*Datura stramonium* L	POONCH-39	Datura	Herb	Whole plant	Rodents, rabbits, canine	Central nervous system	Tachycardia, decreased bowel activity, hyperpyrexia, urinary retention, and neurological disorders with ataxia, impaired short-term memory, disorientation, confusion, hallucinations and finally death
Solanaceae	*Hyoscyamus niger* L	POONCH-40	Henbane	Herb	Whole plant	Livestock, fish, wild animals	Hepatic, Central nervous system	Dryness of throat, intense thirst, headache, nausea, vomiting, giddiness, stomach-ache, trembling of limbs, convulsions and finally death
Solanaceae	*Solanum surattense* Burm.f	POONCH-41	Neeli kandiari	Herb	Berries	Rodents, cattle, sheep, horse	Hepatic	Piloerection, swelling of the face, abdominal pain, drowsiness, and severe itching
Taxaceae	*Taxus baccata* L	POONCH-46	Yew	Tree	Leaves	Cattle, horses	Vital organs	Pulmonary congestion and edema, trembling, rapid breathing
Thymelaeaceae	*Daphne oleoides* Schreb	POONCH-43	Mushk	Shrub	Leaves	Domestic animals	Reproductive systems	Infertility, loss of pregnancy
Ulmaceae	*Celtis australis* L	POONCH-44	Patri	Tree	Leaves	Rodents, rabbits, canine		Regular consumption of leaves causes weakness and an increase in body temperature in animals
Verbenaceae	*Lantana camara* L	POONCH-45	Panjpholi	Shrub	Flowers	Cattle, small ruminants, horses, pigs, rabbits, rodents, and canine	Hepatic, renal, central nervous system, and muscles	Hepatotoxicity, chronic cholestasis, piloerection, photosensitization, intrahepatic cholestasis

### Toxic plant part (s) and most susceptible organs/systems

**FIGURE 5:**
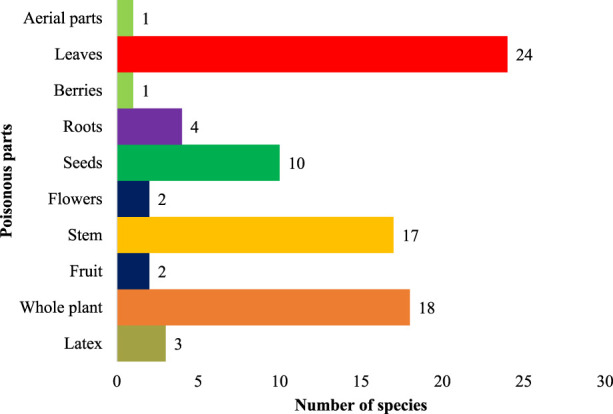
Percentage of poisonous plant parts.

**FIGURE 6:**
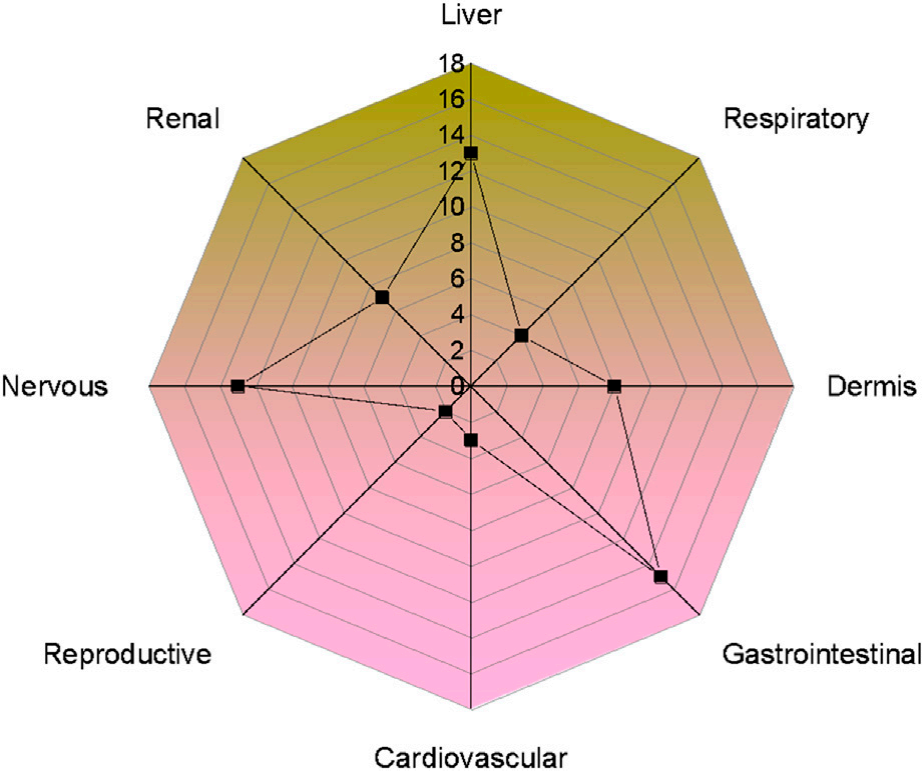
Number of poisonous plants causing different system/organ disorders in livestock of the study area.

Plant toxicity in livestock was observed either in the entire plant or limited to some plant parts (Figure 5). The most toxic plant parts were leaves (24 species, 52.1%), followed by the whole plant (18 species, 39.1%), stem (17 species, 36.9%), seeds (10 species, 21.7%), roots (4 species, 8.7%), latex (3 species, 6.5%), fruit (2 species, 4.3%), flower (2 species, 4.3%), latex (2 species, 5.2%), flowers toxicity (1 species, 2.6%) and berries and aerial parts (1 species each, 2.1%). The most susceptible organ (Figure 6) was the liver, affected by 13 species (28.2%), followed by the gastrointestinal tract, affected by (15 species 32.6%), nervous system (13 species, 28.2%), dermis (8 species 17.3%), renal (7 species, 15.2%), respiratory (4 species, 8.7%), cardiovascular system (3 species, 6.5%), reproductive system (2 species, 4.3%).

### Relative frequency of citations

*N. oleander* showed maximum RFC values (0.66), followed by *L. camara* (0.63), *R. communis* (0.60), *D. stramonium* (0.59), *S. surattense* (0.57), *A. indica* (0.54), *L. cordifolius* (0.53), *P. hysterophorus* (0.50), *E. helioscopa* (0.49), *M. philippinensis* (0.46), *J. adhatoda* (0.43), *D. viscosa* (0.39), and *B. variegata* (0.39) (Table 5).

**TABLE 5:** List of poisonous plants showing Relative Frequency Citation (RFC) and Fidelity Level (FL). RFC shows the citing percentage (FC) of informants for a particular plant species while FL shows the percentage of informants who mentioned a certain poisonous plant for toxicity in livestock in the study area.

Species	FC	RFC	FL (%)
*Achyranthes aspera* L	9	0.13	68.1
*Conium maculatum* L	16	0.23	62.3
*Heracleum canescens* Lindl	19	0.27	71.4
*Justicia adhatoda* L	30	0.43	73.2
*Nerium oleander* L	46	0.66	100
*Alocasia indica* (Lour.) Spach	38	0.54	92.5
*Hedera nepalensis* K.Koch	6	0.09	83.0
*Calotropis procera* (Aiton) W.T.Ation	7	0.10	66.3
*Bidens bipinnata* L	5	0.07	74.6
*Senecio vulgaris* L	4	0.06	70.0
*Ageratum conyzoides* L	18	0.26	76.4
*Xanthium indicum* J.Koenig ex Roxb	10	0.14	54.7
*Parthenium hysterophorus* L	35	0.50	86.4
*Cannabis sativa* L	27	0.39	84.5
*Convolvulus arvensis* L	17	0.24	70.2
*Ipomoea carnea* Jacq	15	0.21	68.1
*Ipomoea purpurea* (L.) Roth	9	0.13	70
*Cuscuta reflexa* Roxb	7	0.10	58.2
*Ricinus communis* L	42	0.60	100
*Euphorbia helioscopia* L	34	0.49	85.1
*Euphorbia heterophylla* L	8	0.11	70.6
*Mallotus philippensis* (Lam.) Müll.Arg	32	0.46	72.1
*Bauhinia variegata* L	27	0.39	70.0
*Dalbergia sissoo* Roxb. ex DC.	6	0.09	60.4
*Lathyrus odoratus* L	4	0.06	55.7
*Melilotus indicus* L	11	0.16	69.1
*Hypericum perforatum* L	19	0.27	83.2
*Melia azedarach* L	21	0.30	81
*Leucaena leucocephala* (Lam.) de Wit	14	0.20	79
*Argemone mexicana* L	15	0.21	80.1
*Leptopus cordifolius* Decne	37	0.53	84.1
*Anagallis arvensis* L	14	0.20	73.1
*Digitalis purpurea* L	20	0.29	76.3
*Clematis grata* Wall	12	0.17	66.0
*Aconitum heterophyllum* Wall. ex Royle	17	0.24	84.1
*Caltha palustris* L	9	0.13	70.6
*Ranunculus arvensis* L	9	0.13	68.3
*Datura innoxia* Mill	18	0.26	77.1
*Datura stramonium* L	41	0.59	95.1
*Hyoscyamus niger* L	12	0.17	72.5
*Solanum surattense* Burm.f	40	0.57	92.9
*Dodonaea viscosa* Jacq	27	0.39	74.1
*Daphne oleoides* Schreb	7	0.10	62.1
*Celtis australis* L	7	0.10	70.4
*Lantana camara* L	44	0.63	100
*Taxus baccata* L	15	0.21	80.3

### Fidelity level (FL)

The highly poisoned species were *N. oleander* (Fl, 100)*, L. camara* (Fl, 100)*, R. communis* (Fl, 100), *D. stramonium* (Fl, 95.1)*, S. surattense* (Fl, 0.9), *A. indica* (*Fl*, 92.5), *P. hysterophorus* (*Fl*, 86.4)*, E. helioscopa* (*Fl*, 85.1)*, L. cordifolius* (*Fl*, 84.1)*, D. viscosa* (*Fl*, 74.1)*, M. philippinensis* (*Fl*, 72.1)*, J. adhatoda* (*Fl*, 73.2), and *B. variegata* (*Fl*, 70.0) (Table 5).

### Priority ranking test

In this test (Table 6), 15 selected key informants were asked to rank the plants based on their toxicity in the study area, following Martin (1995) and Alexiades (1996). The top poisonous plants were selected based on informant scoring, which depends on the toxicity of the plants, and their toxicity (LD_50_) was later confirmed from the literature survey and by performing acute toxicity assays. The total number of informants used was 15, and informants were asked to assign a score out of 100 according to their preference for toxicity. The top-ranked poisonous plant was *L. camara* (91), followed by *R. communis* (86), *N. oleander* (82), *P. hysterophorus* (79), *D. stramonium* (75), *S. surattense* (68), *L. cordifolius* (65), *M. philippinensis* (68), *E. helioscopa* (61), *A. indica* (55), *J. adhatoda* (44), *D. viscosa* (41), *and B. variegata* (39).

**TABLE 6:** List of highly poisonous plants showing informant scores and their ranking obtained from priority ranking test and acute toxicity assessment of these plants through a Limit test. In the Limit test, 0, 1, 2, and 3 show the number of mortalities in rats. Tested LD_50_ shows the lethality of these plants in rats obtained through the current experimental study, which is further compared with LD_50_ reported in different literature.

Plants	Informants score out of 100 (n = 15)	Ranking	Limit test		Tested LD_50_	Reported LD_50_
			2,000 mg/kg	5,000 mg/kg		
*Lantana camara*	91	1st	0	3	4,500 mg/kg	25–50 mg/kg
*Ricinus communis*	86	2nd	0	2	4,200 mg/kg	<20 mg/kg
*Nerium oleander*	82	3rd	0	2	4,000 mg/kg	1,537 mg/kg
*Parthenium hysterophorus*	79	4th	0	2	4,900 mg/kg	<2,000 mg/kg
*Datura stramonium*	75	5th	0	1	4,700 mg/kg	4,000 mg/kg
*Solanum surattense*	68	6th	0	1	>5,000 mg/kg	>2,000 mg/kg
*Leptopus cordifolius*	65	7th	0	0	>5,000 mg/kg	>2,000 mg/kg
*Mallotus philippensis*	61	8th	0	2	>5,000 mg/kg	>2,000 mg/kg
*Euphorbia helioscopia*	55	9th	0	0	>5,000 mg/kg	1,211–2,000 mg/kg
*Alocasia indica*	49	10th	0	0	>5,000 mg/kg	>2,000 mg/kg
*Justicia adhatoda*	44	11th	0	0	>5,000 mg/kg	5,000 mg/kg
*Dodonaea viscosa*	41	12th	0	0	>5,000 mg/kg	1,250 mg/kg

### The LD_50_ of various plants, according to literature survey

As depicted in Table 6, *R. communis,* was the most toxic plant in the light of literature, out of 12 plants that were also tested for toxicity in the current study, with LD_50_ of less than 20 mg/kg. This plant showed different symptoms in livestock, such as general body weakness, loss of appetite, difficulty in breathing, mild fever, abdominal pain, cramps, piloerection, miscarriage, neurological issues, depression, acute purgation, and sudden diarrhea. *L. camara*, on the other side, with LD_50_ 157.37 mg/kg, caused liver damage, bile flow obstruction, hair erection, light sensitivity, and accumulation of bile in the liver. *N. oleander* with an LD_50_ 157.37 mg/kg causes bulging of the eyeballs, stomach pain, salivation, emesis, motion, piloerection, irregular heartbeat, weakness, and sluggishness. *E. helioscopa,* with LD_50_ of 1,211 mg/kg, caused acute dermatitis when animals encounter milky sap, piloerection, frothing in the mouth and throat, purgation, weight loss, and *D. viscosa* LD_50_ 1,250 mg/kg caused nausea, vomiting, and shivering in animals. The rest of the plants showed LD_50_ > 2,000 mg/kg, which, according to OECD 423, is less toxic.

### Limit test for determination of plant LD_50_

**FIGURE 7:**
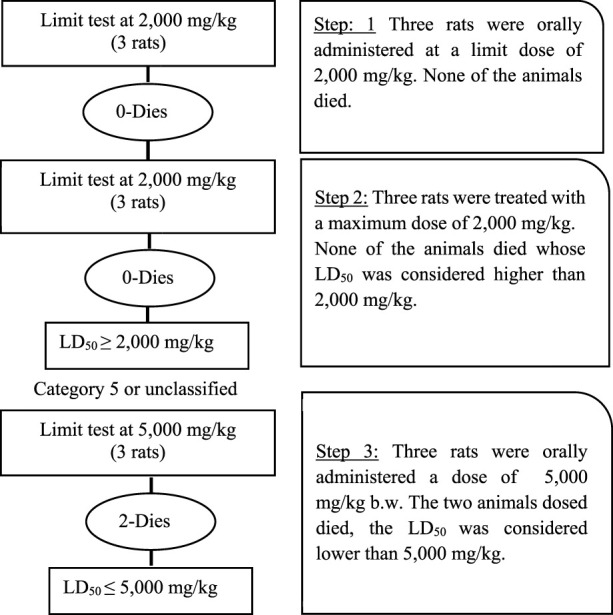
Systematic overview of acute toxicity in rats determined through limit test showing number of rats, dose concentration, and lethalities.

The female Wistar rats orally administered ethanol extract at a dose of 2,000 mg/kg body weight showed no signs of toxicity or mortality for all 12 plants tested for toxicity (Table 6; Figure 7). However, when a 5,000 mg/kg body weight dose was applied, signs of toxicity, mortality, and a significant reduction in body weight were found in various selected poisonous plants, namely, *N. oleander*, *R. communis*, *L. camara*, and *D. stramonium*. Moreover, the mortality of animals was observed within 10–24 h with severe clinical manifestations, i.e., anorexia, water intake, piloerection, dyspnea, salivation, tachypnea, constipation, diarrhea, tremors, itchy nose, and dullness. The severe toxic effect caused by *N. oleander* was followed by *R. communis*, *L. camara,* and *D. stramonium,* but other plants showed moderate to mild clinical manifestations. For the determination of the exact LD_50_ of each plant, extracts were tested through the Up-and-Down method. The LD_50_ for poisonous plants was found to be 4,000, 4,200, 4,500, and 4,700 mg/kg for *N. oleander*, *R. communis*, *L. camara*, and *D. stramonium*, respectively (Table 7).

**TABLE 7:** Clinical symptoms of toxicity in different groups of rats treated with control, sham control, and poisonous plant extract. Where (−) shows no symptoms, (+) shows mild symptoms, (++) shows moderate symptoms, and (+++) shows severe symptoms.

Groups	Anorexia	Water intake	Piloerection	Dyspnea	Salivation	Tachypnea	Constipation	Diarrhea	Tremor	Itching nose	Crowed	Dullness
Control	-	-	-	-	-	-	-	-	-	-	-	-
Sham control DMSO	-	-	-	-	-	-	-	-	-	-	-	-
*Nerium oleander*	++	+	+++	++	+	++	-	++	++	++	++	++
*Ricinus communis*	+	+	++	++	-	++	+	-	+	+	+++	+
*Lantana camara*	++	+	+++	++	-	++	+	-	++	++	++	++
*Solanum surattense*	+	+	+	+	+	+	+	-	+	+	+	+
*Datura stramonium*	+	+	+	+	+	+	+	-	+	+	+++	+
*Leptopus cordifolius*	-	-	-	-	-	-	-	+	-	+	+	+
*Euphorbia helioscopia*	-	-	-	-	-	+	-	-	-	+	+	+
*Parthenium hysterophorus*	++	+	+	+	+	++	-	+	++	++	++	++
*Alocasia indica*	+	+	+	+	-	+	+	-	+	+	+	+
*Mallotus philippensis*	+	+	+	+	-	+	+	-	+	+	+	+
*Justicia adhatoda*	+	-	+	-	-	+	-	-	-	+	-	-
*Dodonaea viscosa*	+	-	+	-	-	+	-	-	-	+	-	-

### Pearson’s correlation

**FIGURE 8:**
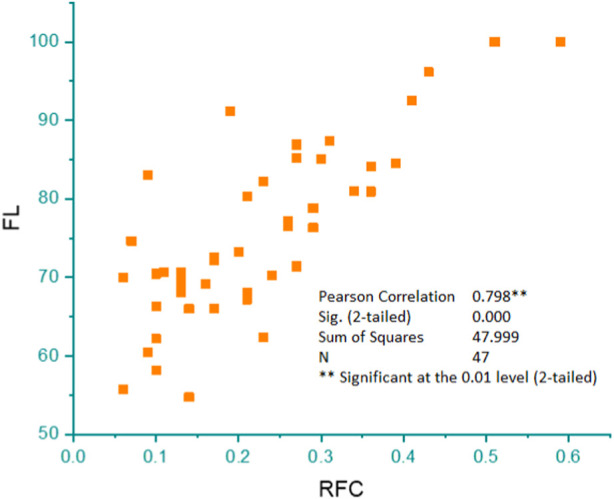
Pearson’s correlation showing the linear relationship between the percentage of informants who mentioned a certain poisonous plant species (FL) and the citing percentage of informants for a particular plant species (RFC).

Pearson’s correlation coefficient was computed to assess the relationship between the level of reported fidelity and RFC. The outcome reveals a substantial correlation between the FL and RFC variables, with a correlation coefficient of r = 0.9 (Figure 8).

## Discussion

In our ecosystem, plants are essential for food, shelter, and aesthetic beauty (Saha et al., 2022). Nevertheless, not all plants are harmless when they interact with animals. Certainly, many plant species contain toxic compounds that can cause animals from mild irritation to severe illness or even death (Koshariya et al., 2023). Understanding plant toxicity in animals is critical for veterinarians, cattle holders, nomads, and wildlife managers to ensure the health and safety of both domestic and wild animals (Gupta, 2012; McKenzie, 2020). Our study area selection was motivated by the rich plant biodiversity and the lack of research on poisonous plants and their toxicity to livestock. As far as we know, no research has been conducted on plant phytotoxicity in District Muzaffarabad and its surroundings. A comprehensive study on the phytotoxicity of plants in this region is extremely important because this area has a significant number of small ruminants compared to other parts of Azad Jammu and Kashmir. It is crucial for farmers to be able to identify which plants are toxic for livestock and to take the necessary precautions to avoid them in order to prevent livestock from consuming them.

### Demographic dynamics

In this study, 70 key informants interviewed were selected from both genders. Our informant selection is counter to the majority of ethno-veterinary studies, where men predominate. In the study area, women typically keep and care for animal herds, which can be explained by the ethnotoxicology study’s nature, cultural differences, and traditional roles in which women are responsible for household wellbeing and health. (Kefifa et al., 2020). The majority of informants were between the ages of 45 and 60 years. Age thus guarantees both a wealth of knowledge and expertise in such cases. These findings are consistent with those of Dansou et al. (2021) and Ouachinou et al. (2017), who reported that informants over the age of 40 are much more experienced. Kefifa et al. (2020) and Houndje et al. (2016) also revealed that most respondents were between 41 and 50–59 years old. This indicates that older people serve as sources of ethnomedical or ethno-veterinary wisdom, preserving knowledge of veterinary herbal medicine or toxicology over the years.

### Plant diversity

This study reported 46 poisonous plants from 23 families and 38 genera. Asteraceae, Fabaceae, Ranunculaceae, Solanaceae, Euphorbiaceae, and Convolvulaceae were the most dominant families. Herbs were the most prevalent form of life, followed by shrubs and trees. Asteraceae has many species, including *Bidens bipinnata*, *Senecio vulgaris*, *Ageratum conyzoides*, *Xanthium indicum*, and *P. hysterophorus*. Asteraceae has been reported as a dominant plant family by many researches (Sema et al., 2018; Kefifa et al., 2020). There is a significant variation in floristic diversity within plant families depending on climate and study area (Dansou et al., 2021). This difference could also be explained by the diversity of socio-cultural clusters that differ from one nation to another since the utilization of natural plant resources for healing intentions might be linked with the knowledge of these socio-cultural groups. The Asteraceae, Solanaceae, Fabaceae, and Euphorbiaceae stand out as some of the most significant families of poisonous plants. Many researchers have previously documented the plants in these families as primarily responsible for plant poisoning (Wink, 2013; Parihaar et al., 2014; Diaz, 2015). Toxins such as alkaloids such as atropine, hyoscyamine, and scopolamine (Baloch et al., 2017), cynogenic glycosides like anthocyanins and anthrachinone (Randrianarivo et al., 2014; Serrano, 2018), saponin including both terpenoid (Randrianarivo et al., 2014) and steroids (Umair et al., 2017; Dusemund et al., 2018), and cyanogenic glucosides (Junior et al., 2022) make these families toxic.

### Toxic plant parts and their toxicity

In the present study, phytotoxicity was confined to the entire plant or limited to some parts. The most toxic plant parts were the leaves, followed by the whole plant. The most susceptible systems to plant toxicity were the gastrointestinal tract, affected by 15 species; the liver affected by 13 species, and the nervous system affected by 13 species. It has been widely shown that leaves can be used for both therapeutic and grazing reasons. Leaves are metabolically dynamic plant parts (Alexiades, 1996). This is due to the fact that leaves are extremely nutrient-rich plant components that animals can readily chew through or swallow, unlike seeds and fruits (Matsuura et al., 2016). According to our research, most of the toxicity in livestock was produced by plants rich in cyanogenic chemicals, glycosides, saponins, and alkaloids. Animals exposed to alkaloids experience severe toxicity, which damages their liver and affects their neurological system (Adibah and Azzreena, 2019). In the majority of cases, they are lethal and cause depression, miscarriage, shivering, nausea, and immobility (Langford and Boor, 1996; Vetter, 2000; Baloch et al., 2017). Conversely, glycosides can induce paralysis, agitation, weakness, burning, tremors, and vision loss (Nguyen et al., 2020; Kharchoufa et al., 2021; Chen et al., 2023). Saponins can cause skin inflammation, liver issues, agitation, burning of skin, and necrosis, leading to kidney failure in livestock (Tadesse et al., 2017). Poisonous herbs and shrubs pose the greatest threat to grazing animals (Baloch et al., 2017; Bricarello et al., 2023). As in our case, both life forms were commonly available in the study area for cattle, whereas tree forage was typically offered to them by herders under food shortage.

### Relative frequency of citation and fidelity level of plants

*N. oleander* showed maximum relative frequency of citation (RFC) values (0.6), followed by *L. camara* (0.6), *D. stramonium* (0.4), *R. communis* (0.6), *S. surattense* (0.5), *A. indica* (0.5), *L. cordifolius* (0.5), *P. hysterophorus* (0.5), *E. helioscopa* (0.4), *M. philippinensis* (0.4), *J. adhatoda* (0.4), *Dodonaea viscosa* (0.3), and *B. variegata* (0.3). For instance, if a researcher wanted to assess the phytotoxicity potential of these plants, they might choose to plant *N. oleander*, *L. camara*, or *R. communis* due to their high RFC values. Alternatively, a gardener looking for low-maintenance landscaping options might choose *A. indica* or *L. cordifolius*, as they still have relatively high RFC values. However, it is important to note that high RFC values do not always indicate desirable qualities. For example, *P*. *hysterophorus* has a high RFC value but is considered an invasive species in many parts of the world, causing harm to native plants and ecosystems. Similarly, *D. stramonium* is highly toxic and can be dangerous if ingested or handled improperly. Plants with high RFC value had a reputation for being poisonous among the local inhabitants of the region. These plants have the potential to serve as the foundation for subsequent evaluation of the assessment of phytochemical profiles and further toxicological studies (Panter et al., 2011). It is crucial to prioritize the conservation of these plants to ensure the sustainable use of these resources, as they also have therapeutic properties (Mukheriee and Wahile, 2006). Notably, *N. oleander*, *L. camara*, *R. communis*, *D. stramonium*, *Solanum surattense*, and *A. indica* were among the highly toxic species. Plants exhibiting higher FL are considered suitable for further ethno-toxicological research (Bibi et al., 2014). Plant fidelity level shows the degree of reliance and consistency of local communities on specific plant species for various purposes (Chaachouay et al., 2022). It also highlights the cultural, medicinal, and economic significance of plants within a given society (Leonti, 2022). The level of plant fidelity can vary widely among different cultures and regions, depending on factors such as traditional knowledge, the availability of resources, and environmental conditions (Pratama et al., 2021).

### Experimental acute toxicity

The current study aimed to assess the toxicities of poisonous plants in District Muzaffarabad, AJK. To achieve this, Wistar rats were subjected to a limit test following OECD guidelines to determine the toxicity of the plant extracts. At a dose of 5,000 mg/kg body weight, *N. oleander*, *R. communis*, *L. camara,* and *D. stramonium* caused toxicity and mortality. In contrast, other plants showed moderate to mild clinical manifestations. Additionally, mortality of animals was observed within 10–24 h with severe clinical manifestations, i.e., anorexia, water intake, piloerection, dyspnea, salivation, tachypnea, constipation, diarrhea, tremor, itching nose, and dullness.

There is a large diversity in methodology and purposes of various experimental studies whereby the toxicity of plants has been studied. Studies use diverse methods of preparing plant extracts, doses, routes of administration, animal species, etc., which result in variable toxicity reported as LD_50_ of various plants. Bandara et al. (2010) and Ceci et al. (2020) reported that the clinical signs of oleander toxicity are basically the same in all species. For the determination of the exact LD_50_ of each plant through the fine-tuning method, LD_50_ was considered poisonous plants. *N. oleander* with 4,000 mg/kg, followed by *R. communis*, *L. camara*, and *D. stramonium*, were found highly poisonous, indicating that their LD_50_ is less than 5,000 mg/kg body weight for four plants. In the case of the *N. oleander* plant extract, different researchers have reported varying LD_50_ values (Ceci et al., 2020; Shridhar, 2022). The lethal doses of various dried *N. oleander* leaves vary depending on the animal species (LD_50_). Cattle are more susceptible than small ruminants, according to the LD_50_, which is 50 mg/kg for cattle, 110 mg/kg for goats, and 250 mg/kg for sheep (Oryan et al., 1996). The LD_50_ of oleander for mice is 4,000 mg/kg, according to research conducted by others. While several studies came to the semi-quantitative conclusion that 3–10 oleander leaves could be deadly, the LD_50_ of oleander leaves for cattle is 50 mg/kg. According to reports, the LD_50_ of oleander for sheep is 250 mg/kg. However, there is disagreement on the lethal dose of oleander (Shridhar, 2022). According to the toxicity and lethality indicators associated with the type of toxin present in the plant’s aerial parts, the lethal dose of *Thevetia peruviana* for rats and mice is 447 mg/kg body weight (Ea et al., 2021).

Different susceptibilities may be due to experimental conditions and methodological differences. In the case of *N. oleander* plant extract, the LD_50_ values reported by different researchers may have resulted from differences in the experimental conditions and methodology used. For example, the use of different animal species, routes of administration, and types of extracts can affect the LD_50_ values reported. Additionally, differences in the preparation of an extract, like the concentration and purity of the extract, can also contribute to differences in LD_50_ values. Various clinical manifestations, such as diarrhea, abdominal pain, irregular heartbeat, seizures, coma, and death, were observed in rats receiving treatments. According to Prasad et al. (2016), the signs of toxicity in rats that appeared after oral administration of the ethanol extract of the plant’s aerial parts included tremor, dyspnea, tachypnea, severe hypothermia, muscular relaxation, urinary retention, generalized seizures, and death.

In the current study, the LD_50_ of the *R. communis* plant was determined to be 4,200 mg/kg. Similarly, Tewari et al. (2005) reported a rat oral LD_50_ of 4,560 mg/kg for *R. communis.* According to Das et al. (2000), the LD_50_ of *R. communis* was found to be 4,750 mg/kg body weight in rats. Additionally, in disagreement with the current study, Polito et al. (2019) reported LD_50_ value of 2,000 mg/kg for an intraperitoneal administration of a ricin-containing fraction of *R. communis* seed extract in mice. Murade et al. (2021) determined the oral LD_50_ of *R. communis* leaves in mice to be 89.3 mg/kg. The oral LD_50_ of *R. communis* leaves in rats was reported to be 131.1 mg/kg by Riaz et al. (2012).

When administered intravenously, the LD_50_ of ricin in rats is roughly 1.78 mg/kg, and when administered orally, it is roughly 21.8 mg/kg (Worbs et al., 2011). The authors further reported that the rats are particularly susceptible to the toxic effects of ricin, and even small doses can result in severe illness or death. Lethargy, weakness, loss of appetite, diarrhea, seizures, and respiratory failure are all possible signs of ricin poisoning in rats. On the other hand, Sadashiv (2011) reported that mice given an ethanol extract of the aerial parts of the *R. communis* plant did not exhibit significantly different behaviors.

In the present study, the LD_50_ (4,500 mg/kg) was observed for *L. camara*, which is in line with the study of Sharma et al. (2007). On the other hand, Jawonisi and Adoga, (2017) also reported LD_50_ greater than 5,000 mg/kg body weight in the rats. Kumar et al. (2016) reported an LD_50_ value of 2,526 mg/kg for an aqueous extract of *L. camara* leaves administered intraperitoneally in rats. However, Pandeya et al. (2022) reported an LD_50_ value of 2,891 mg/kg for an ethanolic extract of *L*. *camara* leaves administered orally to mice. In the current study, we observed some signs of toxicity such as loss of body weight, tremors, piloerection, diarrhea, and food and water consumption in rats administrated with *L. camara*. Mukherjee et al. (2010) tested the toxicity of *L. camara* leaves in rats and found symptoms of toxicity, such as weight loss, decreased food intake, and altered liver function. The rats also exhibited neurological symptoms like tremors, convulsions, and paralysis. Lantadenes is the toxic principle in *L. camara* leaves (Bevilacqua et al., 2011; Pour et al., 2011). Similarly, Sharma et al. (2007) observed that the main hepatotoxic compound is lantadene A, a pentacyclic triterpenoid.

The LD_50_ for *D. stramonium* was found to be 4,700 mg/kg in the present study. Murtala et al. (2023) determined that the LD_50_ was greater than 2,000 mg/kg. According to Lian et al. (2022), typical lethal doses of Datura seeds ranging from 50 to 100 may have atropine. In the experiment, clinical manifestations such as decreased activity and lethargy, ataxia (loss of coordination), tremors or seizures, salivation and lacrimation (excessive salivation and tearing), mydriasis (dilated pupils), respiratory depression or difficulty breathing, diarrhea, coma, or death were observed (Friedman, 2004). *D. stramonium* could trigger tachycardia, difficulty breathing, convulsions, and a decrease in locomotor activity in the presence of high levels of alkaloids such as atropine, hyoscamine, and scopolamine (Ogunmoyole et al., 2019). S*. surratense* is known to contain toxic compounds such as chaconine and solanine (Parvez et al., 2019). In the current study, symptoms of toxicity, such as gastrointestinal upset, vomiting, diarrhea, and even more severe symptoms, such as seizures and respiratory depression, were observed in our experiment.

In the present study, the LD_50_ of *L. cordifolius* was determined through a limit test and is greater than 5,000 mg/kg. Khan et al. (2018) reported several bioactive compounds from *L. cordifolius,* including cicutoxin, chlorogenic acid, rutin, and quercetin. The limit test results showed that LD_50_ exceeded 5,000 mg/kg. These findings imply that *L. cordifolius* is reasonably safe because it takes a high dose to be fatal. Several toxic substances, including diterpenes, triterpenes, phorbol esters, and ingenol esters, are known to be present in the plant species *Euphorbia helioscopia*, which is a member of the Euphorbiaceae family. The milky sap of the plant contains the diterpene ester known as helioscopinolide, which is the most notable toxic substance in this plant (Morita et al., 2014). According to the study of Aleksandrov et al. (2019), rats at an oral dose of 1,000 mg/kg showed signs of toxicity such as lethargy, decreased appetite, and diarrhea. Our results depicted that *P. hysterophorus* extract administrated in to rats resulted in an LD_50_ of 4,900 mg/kg. According to Narasimhan et al. (1984), *P. hysterophorus* extract has a toxic LD_50_ of 1,500 mg/kg in mice. It contains the active ingredient in Parthenium, a sesquiterpene lactone called parthenin. Parthenin causes animal toxicity, including hepatotoxicity, nephrotoxicity, and neurotoxicity (Khaket et al., 2015). We also reported LD_50_ of *A. indica* greater than 5,000 mg/kg. According to reports, these plants contain glycosides such as arisaematoside, oxalate crystals that can cause mouth pain and diarrhea (Basu et al., 2014). Desai et al. (1988) observed restlessness and hyperactivity, convulsions and tremors, lethargy, depression, reduced food intake, weight loss, diarrhea, and impaired coordination and balance caused by *A. indica*.

The current study also reported *Mallotus philippensis* with LD_50_ greater than 5,000 mg/kg. According to Thakur et al. (2005), this plant at a 2,000 mg/kg dose caused mortality in all the animals within 24 h. In addition, Gangwar et al. (2014) reported that *M. philippinensis* administered orally to mice at a dose of 1,000 mg/kg caused death in 50% of the animals within 24 h.

The different behavior of plant poisoning reported in our experimental and already reported studies can be attributed to plant nature, growth stages, geographic location, environmental conditions, the abundance of species, and the species and breed of livestock are just a few of the variables that can affect livestock variation (Mensah et al., 2019; Griffiths et al., 2021). The complex interactions between the chemical makeup of plants and the metabolic function of animals cause variations in plant toxicity (Jung et al., 2020). Plant species have different levels of toxins that can affect livestock differently. Poisonous plants can be classified by active ingredients (cyanide, nitrate, oxalate, pyrrolizidine alkaloids, glycosides, etc.), target organs and systems (cardiotoxic, hepatotoxic, neurotoxic, teratogenic plants, etc.), or family and genus (Ohlsen et al., 2022).

## Conclusion

It is evident from the present study that poisonous plants pose a serious threat to livestock in Muzaffarabad. Plants with active ingredients such as cyanide, nitrate, oxalate, pyrrolizidine alkaloids, and glycosides resulted in gastrointestinal, neurological, hepatotoxic, and dermatological disorders in livestock. *N. oleander, L. camara, R. communis*, *D. stramonium, S. surattense*, *A. indica*, *L. cordifolius,* and *P. hysterophorus* were the highly poisonous species reported from the study area. However, other species exhibited mild to moderate symptoms. The study also shows different plant poisonings in nature can be explained by the complex interactions between plants and animals, as well as their nature, growth stages, geographic location, environmental conditions, and livestock species. In order to gain a deeper understanding of traditional knowledge related to poisonous plants and their effects on animal health, future research needs to be conducted in appropriate experimental conditions involving relevant livestock species. Cattleholders should also be informed about toxic plant hazards to reduce the risk of poisoning and provided with appropriate management strategies. Establishing diagnostic laboratories equipped with advanced analytical techniques can help in the early diagnosis and treatment of plant-poisoned animals, reducing mortality and economic losses. The findings of this study emphasize the need for continuous plant research and monitoring.

## Data Availability

The original contributions presented in the study are included in the article/Supplementary material, further inquiries can be directed to the corresponding author.
